# Thigh-Worn Sensor for Measuring Initial and Final Contact During Gait in a Mobility Impaired Population: Validation Study

**DOI:** 10.2196/80308

**Published:** 2025-10-30

**Authors:** Thomas Johnson, Janeesata Kuntapun, Craig Childs, Andrew Kerr

**Affiliations:** 1 Department of Biomedical Engineering, University of Strathclyde, 106 Rottenrow, Glasgow, G40NW, United Kingdom, 44 0141 548

**Keywords:** accelerometers, Teager-Kaiser energy operator, stance phase estimation, stroke, thigh-worn sensor, spaciotemporal

## Abstract

**Background:**

Adapting physical activity monitors to detect gait events (ie, at initial and final contact) has the potential to build a more personalized approach to gait rehabilitation after stroke. Meeting laboratory standards for detecting these events in impaired populations is challenging, without resorting to a multisensor solution. The Teager-Kaiser energy operator (TKEO) estimates the instantaneous energy of a signal; its enhanced sensitivity has successfully detected gait events from the acceleration signals of individuals with impaired mobility, but has not been applied to stroke.

**Objective:**

This study aimed to test the criterion validity of TKEO gait event detection (and derived spatiotemporal metrics) using data from thigh mounted physical activity monitors compared with concurrent 3D motion capture in chronic survivors of stroke.

**Methods:**

Participants with a history of stroke(n=13, mean age 59, SD 14 years), time since stroke (mean 1.5, SD 0.5 years), walking speed (mean 0.93_*ms*_^_−1_^ , SD 0.38 m/s) performed two 10m walks at their comfortable speed, while wearing two ActivPAL 4+ (AP4) sensors (anterior of both thighs) and LED cluster markers on the pelvis and ankles which were tracked by a motion capture system. The TKEO signal processing technique was then used to extract gait events (initial and final contact) and calculate stance durations which were compared with motion capture data.

**Results:**

There was very good agreement between the AP4 and motion capture data for stance duration (AP4 0.85s, motion capture system 0.88s, 95% CI of difference −0.07 to 0.13, intraclass correlation coefficient [3,1]=0.79).

**Conclusions:**

The TKEO method for gait event detection using AP4 data provides stance time durations that are comparable with laboratory-based systems in a population with chronic stroke. Providing accurate stance time durations from wearable sensors could extend gait training out of clinical environments. Limitations include ecological and external validity. Future work should confirm findings with a larger sample of participants with a history of stroke.

## Introduction

Rehabilitation improves recovery after stroke, with better outcomes when applied intensively and tailored to individual needs [1-4]. Wearable technology has the potential to support increased self-managed rehabilitation by providing performance feedback during everyday activities such as walking in the community but needs to be accurate [5].

As an important feature of independent living, the recovery of walking ability is a major rehabilitation goal [6]. Reduced walking ability is common after stroke, particularly in hemiplegic stroke, which causes specific gait impairments such as reduced stance time duration on the hemiplegic side [3,7]. Speed is the most widely reported and clinically accessible metric in stroke rehabilitation, used as a global measure of mobility recovery [8,9]. Speed alone, however, does not provide meaningful information on the underlying impairments needed to inform effective rehabilitation interventions [10]. One metric that could provide this insight and is clinically relevant and sensitive to change, is stance duration symmetry [11-13]. Measuring this metric during everyday rehabilitation is problematic but achievable with wearable sensors; however, such an approach should consider measurement burden and potential Hawthorne effects [14]. Integrating these metrics into existing systems, for example physical activity monitors, may be a more acceptable approach.

One well-established wearable device, designed for measuring free-living physical activity, is the ActivPAL4+ (AP4) activity monitor (PAL technologies), which is a uniaxial accelerometer attached to the anterior thigh, using proprietary algorithms to measure physical activity and posture of a healthy and impaired populations within free-living environments [15]. This includes standing, sitting, walking (durations and transitions) and measurements of stepping cadence, step counts and energy expenditure [16]. The AP4 has good validity for measuring walking bouts at normal walking speeds, intraclass correlation coefficient [ICC (2,1)=0.78] when compared to direct observation [15,17-19].

Many algorithms and analysis techniques have been developed to measure gait parameters from wearable sensors, from frequency domain metrics to more complex approaches that calculate joint angular displacements [20,21]. To detect gait events specifically, most approaches have used peak detection algorithms and zero crossing techniques, but these have not been found to be robust [22-24]. Gait event detection using the Teager Kaiser Energy Operator (TKEO) with AP4 data has been attempted before with Huntington’s disease, to determine initial (IC) and final contact (FC) events, with the resulting stance phase calculation consistently underestimated (0.08 s), compared to video analysis [25,26]. While this technique appears promising from tests in healthy and impaired participants populations data, there is a need to test with a hemiplegic population post stroke who stand to benefit from the enhanced gait rehabilitation, that could be enabled by the feedback from this approach.

The aim of this study, therefore, was to test the concurrent validity of a thigh-mounted physical activity sensor (AP4) for measuring the stance phase duration of hemiplegic gait, with a 3D motion capture system details acting as the gold standard measure. The hypothesis was that the AP4 would have acceptable levels of concurrent validity through an ICC (3,1) greater than 0.75, and a low (<0.1 s) absolute difference between the two systems experience.

## Methods

### Recruitment

The data were collected as part of a larger rehabilitation trial.

### Study Design

Concurrent validation of an accelerometer-based system (AP4, PAL technologies, Glasgow, UK) was performed against 3D motion capture (Vicon) for measuring stance phase duration in participants of hemiplegic stroke.

### Data Capture

Data from the AP4 and motion capture system were captured concurrently from participants with hemiplegic chronic stroke during two 10m walks in a gait laboratory. An AP4 (43 mmx26 mmx5 mm) was attached to the anterior surface of each thigh (ie, hemiplegic and nonhemiplegic side) with the acceleration data sampled at 50Hz. Marker clusters (Pulsars, Vicon) were attached to participants at three locations (posterior pelvis, lateral malleoli) using Velcro straps and tracked with 37 cameras (Viper, Vicon) sampling at 120 HZ. The 3D trajectory data for the clusters was captured using commercial software (Evoke, Vicon) and processed with a customized Python script (version 3.13.2, Python Software Foundation).

### Stance Time Duration Calculation

Stance time durations were calculated from the motion capture data using a coordinate-based algorithm, modified to use ankle-placed cluster markers [27]. For the acceleration data, Teager and Kaiser [28] developed an algorithm that used the amplitude and frequency of a signal to discern its energy. This algorithm is defined as:


(1) $$\Psi \left[x\left(t\right)\right]=\dot{x}\left(t\right)-x\left(t\right)\ddot{x}\left(t\right)$$

with $\Psi \left[x(t)\right]$ equating the energy of the signal, x, at time, t. $\dot{x}(t)$ and $\ddot{x}\left(t\right)$ denoting the first and second derivative of the signal, x, respectively. The discrete time variant is required for determining specific gait events. To obtain this, a 3-sample symmetric difference is calculated, approximating the first and second derivatives. For a discrete one-dimensional accelerometer signal, $x_{n}$, the TKEO signal, $\varphi_{n}$, is obtained through equation (1):


(2) $$\varphi_{n}=\frac{\left[2x_{n}^{2}+{\left(x_{n+1}-x_{n-1}\right)}^{2}-x_{n}\left(x_{n+2}-x_{n-2}\right)\right]}{4T_{s}^{2}}$$

**Figure 1.**
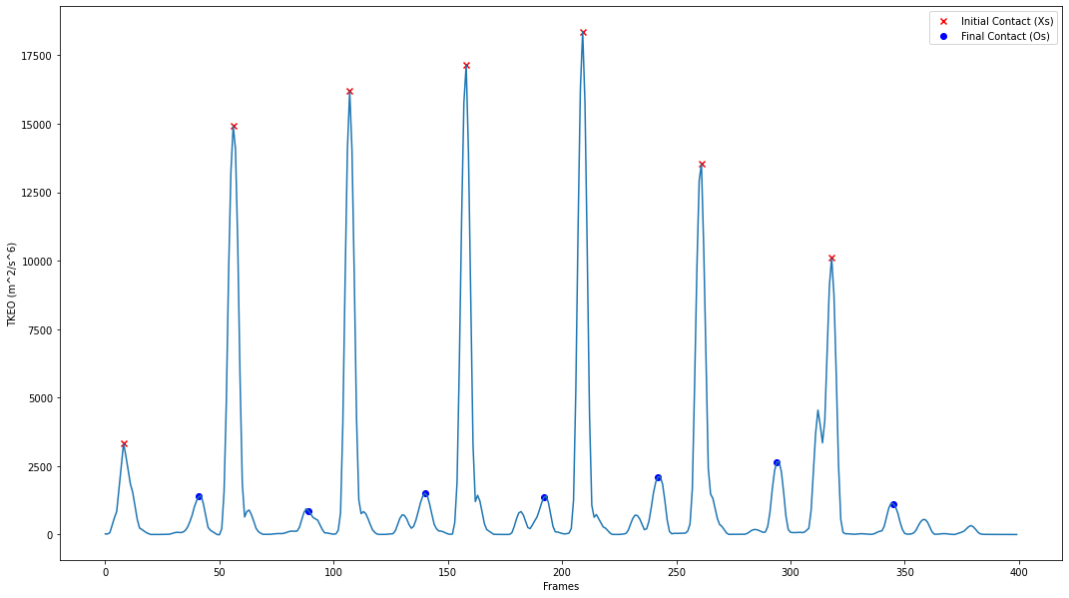
Example TKEO output featuring IC and FC locations. Each large peak in anterior-posterior (AP) acceleration corresponds to IC, red crosses. Processing removed these peaks and calibrated the surrounding regions. After further processing, the largest remaining peaks correspond to FC, blue circles. AP: ; IC: initial contact; FC: final contact; TKEO: Teager-Kaiser energy operator.

Flood et al [26] used equation (2) to amplify accelerometer signal transient features for gait event detection. The TKEO, a nonlinear energy-tracking operator, is considered effective in amplifying sudden changes in signal energy. This allows for identifying gait-related events, such as IC and FC, as seen in Figure 1.

An initial high-pass filter, at 0.5Hz via a 4th-order Butterworth filter is used on the anterior-posterior (AP) acceleration signal of the thigh-mounted sensor. Equation (2) is then applied to obtain $\varphi_{n}$. Once $\varphi_{n}$ was obtained, the processing of the signal was conducted in the same fashion as Lozano-García et al [25], but distinguished by using the constraints and calibration discussed by Flood et al [26].

Applying the TKEO technique meant that the AP4 could remain in its recommended location on the anterior thigh, enabling collection of physical activity parameters using the AP4’s proprietary algorithms [15], as well as collection of stance duration data.

### Data Analysis

A Kolmogorov-Smirnov test was conducted to determine the normality of the difference between the AP4 and Evoke cluster marker system (ECMS). A two-sample *t* test was then used to determine whether the AP4 was statistically different from that of the ECMS when measuring stance times. A Bland-Altman plot and 95% CI of the limits of agreement (LOA) were used to compare the AP4 to the ECMS. A two-way mixed-effect, absolute agreement, single-measures ICC (3,1) was calculated to determine absolute agreement between the AP4 and ECMS.

### Ethical Considerations

This study received ethical approval from Strathclyde University Ethics Committee (UEC25/23: Kerr) and is a registered clinical trial (NCT06787768) [29,30]. All participants of the main trial were invited to take part in this substudy. All participants provided informed consent before their involvement in the study. Participants did not receive compensation, and all data were anonymized.

## Results

The Montreal cognitive assessment (MoCA) is a screening instrument that evaluates general mental capabilities, such as visuospatial abilities, executive functions, and orientation to time and space. The MoCA is rated between 1 and 30, with increasing score dictating better cognitive ability [31]. The functional ambulation category (FAC) evaluates walking ability in 6 levels, with a score of 0 defining no ability to walk, or requiring the help of 2 physiotherapist, and a score of 5 defining full capability to walk independently, including stairs [32]. The Rivermead mobility index (RMI) is an outcome measure used to assess mobility after stroke, rated between 0 and 15, with increasing score indicating better mobility [33] (Table 1).

The AP4 (mean stance time=0.85) saw consistent underestimation in comparison to the ECMS (mean stance time=0.88). Despite this, both methods had a high agreement for stance time measurement (T=0.61, *P*=.54, 95% CI for difference=−0.07s, 0.13s, ICC [3,1]=0.79). The differences between the AP4 and ECMS were shown to be within a standard distribution (*D*=0.11, *P*=.10).

**Figure 2.**
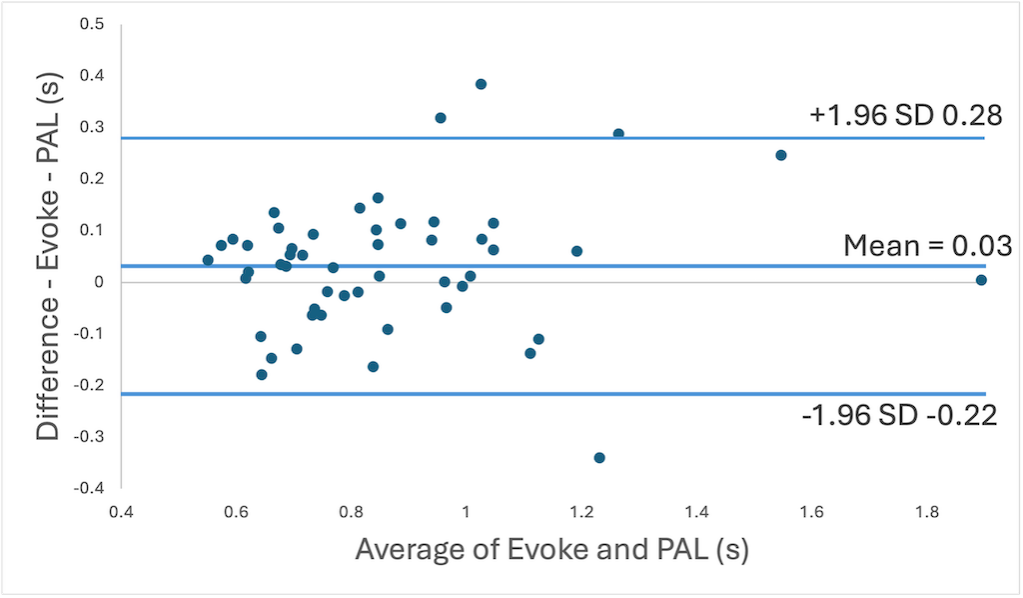
Bland-Altman plot comparing Evoke motion capture system stance time measurement using the Zeni technique with the AP4 using the Teager-Kaiser energy operator (TKEO) measurement technique. The X-axis shows the mean of the two measurements and the Y-axis shows the difference of the two measurements. The central line represents the mean difference with the outer lines representing 95% limits of agreement.

The Bland-Altman plot (Figure 2) displays a consistent spread between the techniques during the performed stroke gait which ranged between 0.49 and 1.90. This shows close agreement between the AP4 and ECMS, irrespective of the value. A bias of 0.03 seconds is reported, suggesting excellent accuracy of the AP4 in comparison to ECMS. The LOA were 0.28s and −0.22s, which contain the 95% of the recorded datapoints. This indicates good agreement between the AP4 and ECMS.

**Table 1.** Participant characteristics of validation study.

Characteristics	Participants (N=13)
Age (years)	30‐78
Aphasia, n	5
Years since stroke, range	1‐13
MoCA^a^ (1-30), range	16‐30
FAC^b^ (0‐5), range	1‐5
Walking speed, range (m/s)	0.06‐1.39
RMI^c^	9‐15

a MoCA: Montreal cognitive assessment.

b FAC: functional ambulation category.

c RMI: Rivermead mobility index.

## Discussion

This study aimed to test the concurrent validity of detecting gait events (ie, initial and final foot contact) and the derived metric—stance phase duration—from thigh acceleration data analyzed with the TKEO, compared with a gold standard 3D motion capture (Vicon) in chronic survivors of stroke. The excellent accuracy (bias=0.03 s) were similar to Flood et al (IC error=0.01s, FC error=0.02 s) [26]and Lozano-García et al (bias=0.08 s) [25] . In the context of a typical stance duration (nonparetic =1.01 s), the reported difference of 0.03 s (2 sampling frames) represents a 2.97% difference, with performance comparable to other commonly used methods of wearable sensor gait parameter measurement, collected by Pacini Panebianco [34] et al. The reported bias of 0.03 seconds could be considered clinically unimportant. A study by Wang [35] et al denoted stance time averages and variability from survivors of stroke at different gait speeds. They noted 0.90 (SD 0.34) seconds and 1.01 (SD 0.41) seconds for the paretic and nonparetic sides, respectively. The magnitude of the bias, therefore, will have little effect on the readings that the AP4 will make by using the TKEO technique. These findings should be confirmed with a larger sample that preserves the diverse nature of the current sample (speed ranged between 0.06‐1.39_ms_^_−1_^ ).

This positive outcome opens opportunity to expand the output of wearable sensors currently used as physical activity monitors, to provide feedback on gait parameters (stance duration, symmetry and weight transference) during the rehabilitation of patients with hemiplegic stroke, without increasing measurement burden.

The results should be interpreted in light of the study’s limitations. The testing took place in a laboratory setting, reducing the generalizability of results. This controlled environment may not reflect everyday gait [36,37]. To obtain ecologically valid gait data, there is a need to capture in free-living environments. The sample of participants with a history of chronic stroke means that only a specific subset (chronic) of the population with stroke has been validated for this technique. Future studies are encouraged to include subacute populations who may have more variable gait parameters. The study had a small sample size, limiting the statistical power and potentially not representing the whole population, although the range of walking ability (walking speed 0.06‐1.39_*ms*_^_−1_^ , FAC 1‐5; Table 1) is reassuringly wide.

In conclusion, the AP4 sensor, in conjunction with the TKEO technique, has been validated against a gold standard 3D Motion Capture system, for stance duration measurement in participants with chronic stroke. However, this positive finding is limited by the study’s setting and small sample. Future work should consider a bigger sample and collect gait data in free-living environments. The outcomes of this study could be exploited to enhance the function of wearable sensors, in order to provide the gait parameters valuable for self-rehabilitation after stroke, such as symmetry.

## Abbreviations

AP4: ActivPAL4+
ECMS: Evoke cluster marker system
FAC: functional ambulation category
FC: final contact
IC: initial contact
ICC: intraclass correlation coefficient
LOA: limits of agreement
MoCA: Montreal cognitive assessment
RMI: Rivermead mobility index
TKEO: Teager-Kaiser energy operator
